# Exploring the Delivery and Management of Specialised Post-Diagnostic Care and Support in Young-Onset Dementia: A Cross-Sectional Study

**DOI:** 10.1177/11786329251388775

**Published:** 2025-11-04

**Authors:** Sophie van Westendorp, Cynthia Hofman, Merwin Mortier, Britt Appelhof, Paula Gerring, Raymond Koopmans, Christian Bakker

**Affiliations:** 1 Department of Primary and Community Care, Radboud University Medical Centre, Nijmegen, The Netherlands; 2 Vilans, National Centre of Expertise for Long-Term Care, Utrecht, The Netherlands; 3 Radboudumc Alzheimer Centre, Radboud University Medical Centre, Nijmegen, The Netherlands; 4 Archipel Landrijt, Knowledge Centre for Specialised Care, Eindhoven, The Netherlands; 5 Groenhuysen, Centre for Specialised Geriatric Care, Roosendaal, The Netherlands

**Keywords:** young-onset dementia (YOD), specialised dementia care, access to post-diagnostic services, healthcare service management, regional disparities, health service delivery

## Abstract

**Introduction:**

The need for tailored services for individuals with young-onset dementia (YOD) is well established. Specialised services exist but regional disparities may hinder timely and appropriate care and support. Yet, a comprehensive overview of such services is currently lacking.

**Objective:**

To examine regional disparities in the delivery, access and management of YOD-specialised services in the Netherlands, revealing service gaps and opportunities for future development.

**Design:**

An exploratory cross-sectional survey (YOD self-scan) was developed and distributed to 39 Dutch healthcare organisations affiliated with a national YOD knowledge infrastructure, ensuring broad geographical representation.

**Methods:**

Quantitative and qualitative data were collected through open- and closed-ended questions between July and August 2023. Descriptive statistics and manifest content analyses were used to assess the delivery and management of YOD-specialised services in terms of utilisation, capacity, accessibility, variety and organisational factors.

**Results:**

A total of 1707 individuals with YOD utilised outpatient services, and 801 received permanent residential care. Service delivery and management varied across regions. Rural organisations reported shorter waiting times, more employees who had received specific YOD training, and higher day care utilisation per organisation. Urban organisations offered a broader range of services and more involvement of local governments. Most organisations (72.4%) reported service gaps, including limited day care options, inflexible residential services, and insufficient support for carers, especially children of individuals with YOD. Also, the need for more innovative services and improved coordination was identified.

**Conclusions:**

YOD-specialised services are underutilised, likely due to unequal accessibility and regional disparities in the delivery and management of services. Addressing service gaps, expanding capacity, and enhancing knowledge exchange are important for equitable, high-quality care. These findings may inform future research and international efforts to improve equitable access and management of specialised dementia services.

## Introduction

Dementia is an irreversible and progressive neurological condition and is considered a public health priority. It is the seventh leading cause of death worldwide, and a major contributor to disability, dependency, and healthcare costs.^1^ Currently, more than 57 million individuals live with dementia worldwide,^2^ including approximately 300 000 in the Netherlands. While most individuals have late-onset dementia (LOD), an estimated 3.9 million people live with young-onset dementia (YOD) worldwide, including 14 000 to 17 000 in the Netherlands.^3^ YOD is defined when onset of the first symptoms of dementia occurs before the age of 65 years.^4^

YOD presents unique challenges. Individuals with YOD have different needs due to their often higher levels of vitality, the heterogeneity in the causes of dementia,^5-7^ and being in a younger phase of life. This disrupts the work,^8^ social and family life of the individuals with YOD and their carers, referring to friends or family who provide unpaid care.^9,10^ Additionally, the time to diagnosis ranges from 3.2 to 5.5 years,^11-13^ resulting in a significant delay in the initiation of appropriate post-diagnostic services,^14,15^ high levels of carer burden and significant unmet needs.^16^

The delivery of appropriate care and support is essential,^16,17^ as it can improve satisfaction with care,^18^ enhance quality of life (QoL),^9,19^ and delay institutionalisation.^9,16^ However, access to YOD-specialised services is often limited due to geographical disparities, insufficient funding, and a shortage of such services.^9,20-23^ This, combined with the relatively low prevalence, the specificity and diversity of required services creates challenges for healthcare organisations in delivering comprehensive, tailored services for YOD within existing infrastructures. International studies consistently report these difficulties, highlighting fragmented service delivery, limited availability of tailored interventions, and variability in access across regions. (Mayrhofer, Mathie, McKeown, Bunn and Goodman, 2018) (Fatima, Mehendale and Reddy, 2022) (van Gils, Rhodius-Meester and Leeuwis, 2023) (Millenaar, Bakker, Koopmans, Verhey, Kurz and de Vugt, 2016) (Sansoni, Duncan, Grootemaat, Capell, Samsa and Westera, 2016)(Giebel, Robertson, Beaulen, Zwakhalen, Allen and Verbeek, 2021) (Bakker, Verboom and Koopmans, 2022) (O’Connell, Crossley and Cammer, 2014).

Similar challenges also exist in the Netherlands. In addition, most people tend to live longer at home instead of being institutionalised,^24^ with the time from onset of first symptoms to transitioning to residential care being almost 9 years in YOD in the Netherlands.^25^ This further influences the requirements in the delivery and management of YOD-specialised services in the Dutch healthcare system, as services increasingly need to be oriented towards supporting home-based care.

In response, the Netherlands has made significant strides in dementia care over the last decade. Since 2014, dementia has been a priority in the national health policy, with specific recognition for YOD from 2021 onwards. The Young-Onset Dementia Knowledge Centre, established in 2013, has been instrumental in developing a national infrastructure for YOD care. Operating under a hub-and-spoke model, the Knowledge Centre (hub) generates and disseminates knowledge, while regional long-term care organisations (spokes) coordinate and deliver YOD-specialised services in collaboration with local partners. In addition to this structure, five academic Alzheimer research centres and the Dutch Alzheimer’s Society contribute to dementia care and research.^22^ The Knowledge Centre has fostered collaboration, facilitated scientific research, supported the development of educational programmes for healthcare professionals involved in YOD care and support, and is recognised by the Dutch government as a key partner in establishing specialised services for YOD.^22^ However, a comprehensive overview of how services are delivered and managed across regions within this national YOD care infrastructure is lacking.

This study addresses that gap by mapping the current landscape of YOD-specialised services in the Netherlands. It identifies regional disparities, service gaps, and organisational challenges, offering insights to inform national and international policy and practice.

## Methods

### Study Design

An exploratory cross-sectional design was chosen as it allows for a broad snapshot of current practices. A survey (named the ‘YOD self-scan’) was specifically developed to gather both quantitative and qualitative data on the delivery and organisational factors in YOD-specialised services.

This study was conducted in the Netherlands as part of the YOD-INCLUDED project, a national research programme aimed at improving diagnostic and post-diagnostic care and support, as well as the connection and transition between different types of care and support throughout the caregiving trajectory, and its accessibility.

### Study Setting and Participants

Organisations were eligible for inclusion if they were affiliated with the Young-Onset Dementia Knowledge Centre. No exclusion criteria were applied, as the aim was to cover as many YOD-specialised services as possible. Therefore, all 39 healthcare organisations affiliated with the Knowledge Centre were invited to participate. Contact was initiated through existing meetings and email outreach between July and August 2023. To maximise response rates, organisations were provided with detailed information on the study’s relevance, participation implications, and potential impact on future YOD-specialised services. Consent from healthcare organisations to participate in the study was obtained from representatives of the participating organisations.

Participating organisations were encouraged to involve relevant team members as the YOD self-scan contained questions across different areas of expertise. Examples of potentially relevant team members included team managers, case managers, client council members, and welfare workers. Two online question-and-answer sessions were organised to support data collection. Tips and insights shared during these sessions were subsequently distributed via email to all organisations.

### Development of the YOD Self-Scan

The YOD self-scan was developed through an iterative process, drawing from a previously performed quantitative questionnaire, recent literature, input from both the board of the Knowledge Centre and end-users, including managers, healthcare professionals, and policy officers (Supplementary Material S1). We piloted the YOD self-scan with two healthcare organisations to ensure its relevance, feasibility, and applicability. Their feedback was obtained via email and telephone interviews, and independently analysed by two research team members (SW, MM). Any disagreements were resolved through discussions with the project team. This collaborative approach ensured that topics addressed in the YOD self-scan were relevant to current challenges in the delivery and organisation of YOD-specialised services. The two pilot organisations were included in the final study, to ensure completeness of the data collection.

### Data Collection

Data collection targeted two domains: (1) the delivery of specialised post-diagnostic services, and (2) the management of these services. These domains consisted of 63 open- and closed-ended questions in total, but not all questions were applicable to every organisation. Participating organisations received sub-questions depending on each organisation’s available services and management structure. Participants had the option to complete the YOD self-scan online or on paper. To validate content, an online session was organised with end-users to discuss results and provide input.

### Delivery of Specialised Post-Diagnostic Services (Domain 1)

Domain 1 captured data on the delivery of common types of specialised services. Common services were defined as widely recognised and routinely available forms of dementia care in the Netherlands, including (YOD-specialised) case management, day care, day care treatment, permanent residential care and supportive services for carers. Uncommon services referred to less standardised forms of dementia care, such as part-time residential care, accommodational residential care and other services not covered by the categories above (Table 1).

**Table 1.** Description of Common and Uncommon Types of Services Available in Dementia Care.

Common types of services		
Outpatient/residential	Type of service	Description
Outpatient services	Young-onset dementia-specialised case manager	A professional, completing an additional education in Young-Onset Dementia-specialised care and support and responsible for coordinating care and providing guidance to both the individual with dementia and their carers
	Day care	(group) activities during day time provided by professionals
	Day care treatment	A combination of activities and treatment during day time provided by professionals
	Services for carers	Support for family members or friends who provide care and support for the person with dementia
Residential service	Permanent residential care	24-hour personal care and support by professionals in a nursing home setting
Uncommon types of services		
Outpatient/residential	Type of services	Description
Outpatient service	Other services	Care or support not defined by common types of outpatient services
Residential services	Part-time residential care	24-hour personal care and support by professionals in a nursing home setting for a part of the time, alternating with periods living at home
	Accommodational residential care	Personal care and support by professionals in a nursing home setting for one night

Where possible, detailed information was collected on: (1) service utilisation, referring to the number of people using each service, (2) service capacity, referring to the number of available places per service within an organisation, (3) accessibility, involving waiting times and maximum distance to a service, (4) service diversity, referring to the type and combination of services delivered by organisations, and (5) identified service gaps, defined as services lacking in the current delivery of services.

### Management of Specialised Post-Diagnostic Services (Domain 2)

Domain 2 focused on organisational factors and management structures relevant to the delivery of YOD-specialised services. Key aspects investigated included: (1) agreements or work protocols, including their presence and level of formal documentation, (2) user participation, such as the involvement of individuals with YOD and their carers in service design and delivery, (3) multidisciplinary teams, referring to a collaboration of different disciplines within an organisation to provide care and support, (4) YOD-specialised staff, defined as the number of employees receiving a specific YOD-training, (5) size of the organisation’s service area, defined by the number of municipalities served by an organisation, and (6) local governments’ involvement in organising YOD-specific activities.

**Figure 1.**
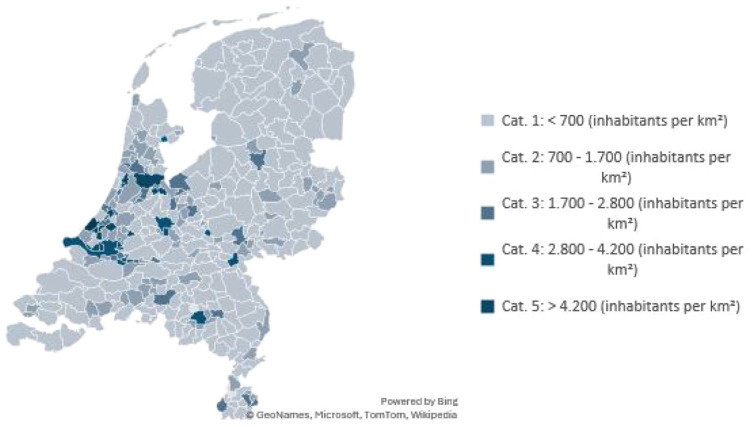
Map with the categorised population density in municipalities in The Netherlands.

Organisations were categorised as urban or rural, based on the population density of the municipality where their headquarters were located. Population density data as of January 2024 was obtained from Statistics Netherlands.^26^ Figure 1 presents a map of Dutch municipalities and their population density, providing context for interpreting the study data and enabling international comparisons. In our study, rural areas were defined as having a population density of less than 700 inhabitants per square kilometers (km^2^), corresponding to the lowest density category (Cat. 1) (Figure 1).

### Statistical Analysis

This study represents an initial, descriptive exploration of YOD-specialised care and support in the Netherlands. As the aim was to obtain a comprehensive overview of service delivery, access, and management rather than to test specific hypotheses or comparing groups, no formal sample size calculation or power analysis was conducted. Instead, we sought to include all 39 organisations affiliated with the Young-Onset Dementia Knowledge Centre, thereby ensuring maximum national coverage and representativeness of YOD-specialised services.

We used descriptive statistics to analyse quantitative data. For skewed distributions, we reported medians, ranges, and interquartile ranges (IQR). Categorical variables were presented as proportions and percentages.

Qualitative data from open-ended questions was analysed using manifest content analysis to identify themes related to service delivery and organisation.^27^ After familiarising with the data, one researcher (SW) identified content that added information influencing service delivery and organisation. This content was categorised thematically where possible and summarised to provide illustrations. Quantitative analyses were conducted using IBM SPSS Statistics, version 29 (IBM Corp., Armonk, NY, USA). No inferential analyses were performed, as the study aimed to provide a comprehensive description rather than test hypotheses. Qualitative content analysis of open-text responses was performed in Microsoft Excel, version 2308 (Microsoft Corporation, 2018).

Missing data originated from non-responding organisations and from responding organisations that were unable to estimate numbers or did not possess the necessary information. Given the extensive scope of the YOD self-scan, which encompasses multiple fields of expertise, the presence of missing data is recognised and assumed to be missing at random. Details on missing data from responding organisations can be found in the Supplementary material.

## Results

### Delivery of Specialised Post-Diagnostic Services

At least 2508 individuals with YOD utilised specialised post-diagnostic services across the 29 participating organisations. Of these individuals, 31.9% (n = 801) received permanent residential care, while 68.1% (n = 1707) accessed one or more types of outpatient services. Among the common outpatient services (Table 1), YOD-specialised case management was most frequently utilised (n = 861). A total of 54 YOD-specialised case managers were employed across 24 organisations, ranging from 1 to 8 employed YOD-specialised case managers per organisation. In terms of capacity, organisations delivered 1022 weekly places for day care treatment, 498 for day care, and 758 rooms for permanent residential care. Capacity varied substantially between organisations, particularly for day care treatment (range: up to 150 places per week).

Access to services differed by type in terms of travel distance and waiting times. The median travel distance was 19 km for day care (range: up to 60 km) and 28 km for day care treatment (range: up to 55 km). The estimated time until people could access a specific service after referral was highest for residential care, with a median of 20 weeks (maximum: 52 weeks). For YOD-specialised case management, the median was 2 weeks (maximum: 30 weeks), for day care treatment 1.5 weeks (maximum: 10 weeks), and 0 weeks (maximum: 4 weeks) for day care.

**Figure 2.**
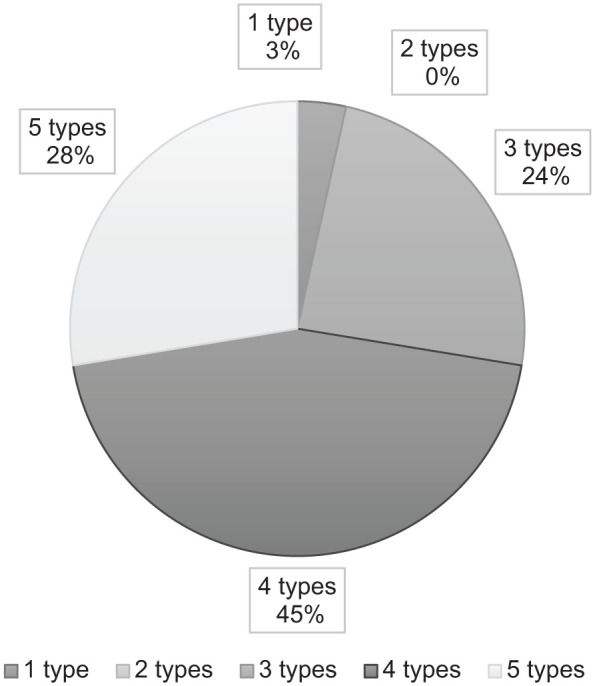
The number of healthcare organisations delivering different types of common specialised services for young-onset dementia (total n = 29 organisations, maximum: 5 common types).

Most organisations delivered a variety of YOD-specialised services. Thirteen organisations (45%) provided four of the five common service types (Figure 2). No organisation offered only residential care, while one delivered exclusively outpatient services. The most frequently used combination included residential care, case management, day care treatment, and carer support. Services for carers varied from peer support groups to digital interventions, such as *Partner in Balance*, an online support programme. In addition to common services, 19 organisations offered uncommon services including 297 rooms for part-time residential care and 14 for accommodational residential care. Fourteen organisations also provided a variety of uncommon outpatient services, such as peer support for children of individuals with YOD, specialised at-home physiotherapy, and consultation clinics for general practitioners managing individuals with YOD living at home.

Overall, substantial variation in service availability, utilisation, types, and waiting time was observed across organisations. Supplementary Material S2 provides further details on these variations, as well as information on missing data for each organisation.

A total of 72.4% of the organisations reported unmet needs in their current services, many of which were provided by other organisations. This included support for individuals living at home (day care groups, peer support, meeting areas, psychoeducational groups, and assistance with finding adjusted work), support for carers (additional services, particularly for children of individuals with YOD), and the availability of more flexible residential care options. Identified gaps included the need for more innovative services, such as home modifications to prolong independent living, day care for individuals receiving permanent residential care, specialised residential units for observation, diagnosis, and treatment in cases where symptoms are unclear and a diagnosis of YOD is suspected, YOD-specialised case management during and after the transition to permanent residential care. Furthermore, organisations noted a need for more YOD-specialised staff, financial support, and integrated care structures, such as collaboration with municipalities or other professionals. Some organisations were actively addressing these issues.

### Management of Specialised Post-Diagnostic Services

Agreements or work protocols regarding the management of YOD-specialised post-diagnostic services existed in 27 of the 28 responding organisations, with 15 fully formalised in written documents. In 19 organisations, covenants were in place for situations where care and support could not be provided. Five organisations referred individuals to other organisations with YOD-specialised services, five maintained a waiting list, and eight used a combination of these approaches or consulted with the individual and their carer to determine the best course of action. Additionally, 19 organisations had agreements regarding access to day care and 18 regarding day care treatment, including arrangements for transportation and maximum travel distances. Most organisations (n = 26) involved individuals with YOD and/or their carers in service design and delivery, as well as in the development of personal care plans.

Multidisciplinary teams were common (n = 27), typically comprising professionals such as elderly care physicians, (healthcare) psychologists, occupational therapists, and physiotherapists (Supplementary Material S3 for details). The frequency with which multidisciplinary teams convened varied from one to four times per month, with 12 organisations meeting four times per month. While 17 organisations considered the availability of YOD-specific training programmes sufficient, 16 reported an insufficient number of staff trained in YOD-specialised care.

Collectively, 28 organisations delivered services across 250 of the total 342 Dutch municipalities. Service areas ranged from 1 to 25 municipalities (median: 7.5). In most municipalities, local government did not organise YOD-specific activities as part of social care (n = 15). Three organisations reported the presence of such activities, while nine indicated that they were in the process of establishing such activities.

### Demographic Disparities in Service Delivery

**Figure 3.**
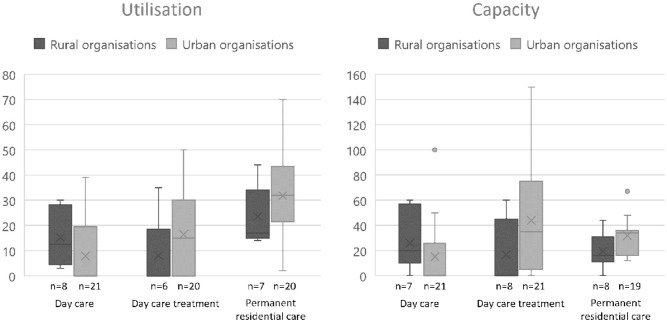
Utilisation and capacity of specialised services for young-onset dementia in rural (maximum n = 8) and urban (maximum n = 21) organisations. Left: utilisation, right: capacity.

The minority of organisations (25.0%, n = 7) were located in rural municipalities, serving 22.0% of the individuals with YOD. Day care services were most utilised in rural organisations, while day care treatment was more utilised in urban organisations (Figure 3).

Capacity of services was comparable between rural and urban organisations, although rural organisations offered more weekly places for day care, whereas urban organisations had greater weekly capacity for day care treatment.

Access to common services was generally more favourable in rural areas in terms of both travel distance and waiting time. On average, individuals in rural areas travelled 17 km for day care, compared to 20 km in urban areas. For day care treatment, the median distance was 24 km in rural areas versus 30 km in urban areas. Waiting times were also shorter in rural areas: 7 weeks for permanent residential care (range: 0-36), compared to 35 weeks in urban areas (range: 0-52). For YOD-specialised case management, the median waiting time was 1 week in rural areas (range: 0-3) and 2 weeks in urban areas (range: 0-30). No median waiting time was reported for day care in either area, but the range was 0 to 2 weeks in rural areas and 0-4 weeks in urban areas. Waiting times for day care treatment were comparable with a median of 2 weeks in rural areas (range: 0-4) and 1.5 weeks in urban areas (range: 0-10).

Rural organisations were less likely to offer all common types of services compared to their urban counterparts (25.0% vs 33.3%). However, both groups reported similar needs to expand their service provision (75.0% in rural vs 71.4% in urban areas).

### Demographic Disparities in Management of Specialised Post-Diagnostic Services

In urban areas, most organisations had agreements or work protocols in place for the management of YOD-specialised care (95.2%), with half of these fully documented. All rural organisations had agreements or work protocols, with 71.4% fully formalised. Urban organisations were less likely to have fully documented agreements regarding access to day care and day care treatment compared to rural organisations, where all agreements in these areas were formalised.

Conversely, urban organisations were more likely to have covenants when care or support could not be provided with a wider range of response strategies. Eight urban organisations referred individuals to other specialised centres, five maintained waiting lists, and seven used alternative approaches, such as offering transitional (non-YOD-specific) dementia care while individuals awaited placement. In contrast, rural organisations did not refer individiduals to other (specialised) centres or placed them on waiting lists, but consistently applied one of the previously mentioned alternative approaches.

Multidisciplinary teams were more common in urban organisations (100.0% vs 85.7%) and teams in urban areas convened more frequently (median: 4 meetings/month vs 1 meeting/month). However, urban teams consisted of fewer disciplines on average (median: 8.0 vs 9.5), though the range was broader, extending to 11 disciplines in urban teams compared to 3 in rural teams. Fewer employees who completed an educational programme for YOD-specialised care and support worked in urban organisations compared to rural ones (median: 11 vs 15).

Urban organisations delivered services to more municipalities than rural organisations (median: 8 vs 5). None of the local governments in rural municipalities had established YOD-specific activities, though 25.0% were in process of initiating them. In contrast, 14.3% of the local governments in urban municipalities had existing YOD-specific activities and 33.3% were in the process of initiating them.

## Discussion

This study provides the first nationwide overview of the delivery and management of specialised post-diagnostic services in YOD in the Netherlands. The findings reveal an underutilisation of specialised services by individuals with YOD, identified gaps within current services and showed disparities in the delivery and management of services per organisation and across regions. These insights outline several key implications for practice and policy by highlighting opportunities for improving and expanding YOD-specialised services in a structured manner. Furthermore, this study serves as a basis for further research into causes of disparities in the delivery of YOD-specialised services and effectiveness of different care models in various geographical contexts.

### Delivery of YOD-Specialised Services

Although the necessity of appropriate services is well recognised^9,16-19^ our study identified that 1707 of the estimated 14 000 to 17 000 individuals with YOD in the Netherlands utilised at least one common outpatient service specifically designed for people with YOD and 801 utilised permanent residential care. This may indicate that a significant proportion of individuals with YOD do not utilise YOD-specialised services, with our results revealing certain barriers in equal accessibility to these services. At least 92 of the 342 Dutch municipalities were not covered by YOD-specialised services. Additionally, the variability in waiting times, reaching up to 52 weeks for permanent residential care, and the large variety in services on offer across participating organisations, with only 24.1% of the organisations providing all common types of services, further illustrates the challenges in ensuring access to certain YOD-specialised services. Also, our study reported a total capacity of 1502 day care and day care treatment places per week, and 758 rooms were available for permanent residential care. While we did not investigate whether this capacity meets actual demand, the mismatch with the estimated number of individuals living with YOD in the Netherlands suggests a potential shortage. Our findings align with international studies, such as the UK, where only 10.7% of individuals with YOD utilised general services,^18^ and 17.6% received early post-diagnostic support.^28^ Similarly, in Australia, only 33.3% of individuals with YOD utilised community-based services, with many declining due to YOD-specific barriers including ineligibility, unaffordability, lack of security, and lack of childcare.^29^ Furthermore, availability is another critical issue with Canadian research reporting a near absence of YOD-specialised services,^30^ and a recent study in Sweden highlighting a shortage of YOD-specialised services.^31^ These findings indicate that ensuring sufficient service provision and improving access to YOD-specific care remains a global challenge.

One essential service is YOD-specialised case management, which plays a crucial role in coordinating care.^18,29,32,33^ Our study found that 54 YOD-specialised case managers were employed across 24 organisations, with a median waiting time of 2 weeks (range: 0-30 weeks). This suggests that while some organisations experience barriers in providing timely YOD-specialised case management, others have managed to deliver timely case management for YOD. However, the absence of data from non-responding organisations limits the generalisability of this finding.

Most organisations identified gaps in order to expand or develop their service provision, including services for children of individuals with YOD. This aligns with recent literature indicating a lack of specific support options, particularly for young family members of individuals with YOD.^31^

Another critical gap was identified regarding the limited availability of structured day care activities, despite the well-documented need for such services.^34,35^ While some organisations and municipalities have introduced innovative initiatives to address this, efforts particularly in social care are not yet widespread. The lack of meaningful daily activities may negatively impact individuals with YOD, reducing their ability to remain engaged in daily life.

### Management of YOD-Specialised Services

Best practices for the organisation of YOD-specialised services include building staff capacity, fostering multidisciplinary collaboration, and involving individuals with YOD and their families in service design.^36^ Previous research has highlighted the importance of working within multidisciplinary teams,^37^ and our study confirms that nearly all participating organisations adopted this approach. Moreover, many organisations facilitated direct involvement of individuals with YOD and their carers in shaping the delivery of care. However, challenges remain as implementation of these organisational structures differed across organisations. A particular challenge concerns the insufficient number of employees with a YOD-specific training. This could not be attributed solely to a lack of YOD-specialised educational programmes.

### Demographic Disparities

Although most Dutch municipalities are classified as rural, our findings indicate that YOD-specialised organisations are less frequently located in these areas. Overall, rural organisations had lower capacity and offered a narrower range of services compared to urban organisations. However, the average capacity and utilisation per organisation for day care services were higher in rural settings, suggesting that available day care services may be relatively more accessible or efficiently utilised. Further research is needed to confirm this hypothesis. Alternative explanations include a better alignment between capacity and utilisation in urban areas or potential barriers in rural areas that reduce service uptake. However, our findings do not support the latter, as access to day care services did not differ between rural and urban regions. Moreover, the availability of YOD-specialised staff was relatively higher in rural areas. Another possibility is more involvement of local governments in organising YOD-specific activities, as shown by our study’s findings in urban areas. Alternatively, rural organisations could compensate for the lack of nearby alternatives, yet our findings do not support this, as they served fewer municipalities on average.

Our assumption that day care services are more favourable in certain rural areas contrasts with previous research, which often reported lower access to YOD services in rural regions. One possible explanation is differences in study designs.^20,38^ For example, Sansoni et al^20^ described limited service delivery and insufficient YOD-specialised staffing in rural areas, yet their conclusions were based on assumptions rather than quantitative data. Similarly, Bauer et al^38^ identified unmet needs and limited service delivery among individuals with dementia in rural regions, but their study lacked quantitative data, and did not specifically focused on YOD or compared rural and urban areas.

A final explanation could be that the Dutch national infrastructure helps mitigate some urban-rural disparities by fostering collaboration between organisations across different regions. The presence of national initiatives, such as the Young-Onset Dementia Knowledge Centre, may contribute to a more even distribution of expertise and resources than in other countries. In addition, certain urban regions may appear better resourced due to the geographical clustering of YOD-specialised organisations, which enables greater service diversity and inter-organisational collaboration, while rural organisations often cover larger areas with fewer nearby alternatives.

### Strengths and Limitations

This study offers a unique, nationwide overview of YOD-specialised service delivery and management in the Netherlands. Its strengths include a high response rate, the inclusion of both quantitative and qualitative data, and the co-development of the YOD self-scan with stakeholders, enhancing the relevance and validity of the findings.

However, several limitations should be noted. First, the exclusion of non-responding organisations and missing data may have led to an underestimation of service utilisation and gaps. However, three of the nine non-responding organisations explicitly stated that they did not provide YOD-specialised services, suggesting limited bias. Second, the sample was limited to organisations affiliated with the YOD Knowledge Centre, potentially excluding other relevant providers. Third, adherence to the instruction to involve multidisciplinary team members in completing the YOD self-scan was not verified, which may have affected data accuracy.

### Implications for Practice and Policy

Our findings underscore the need for continued investment in YOD-specialised services to ensure equitable and high-quality care. Strengthening coordination and funding mechanisms across regions is essential to reduce fragmentation and to ensure accessibility and affordability for this relatively small group with complex care needs. Transparent communication between YOD-specialised organisations about service availability could facilitate more timely referrals and reduce waiting times. To promote equity, specific attention must be paid to rural regions, where limited service availability may restrict timely access to care. Strengthening regional collaboration, supporting outreach initiatives, and ensuring financial compensation for longer travel distances could help reduce these disparities. In addition, municipalities, healthcare organisations, and funding bodies should develop coherent agreements on financing, allowing flexibility to accommodate the heterogeneity of needs across the disease trajectory. Concrete measures could include the introduction of national funding schemes that specifically cover YOD-specialised services, reimbursement of travel costs for individuals and carers in rural areas, and structural investment in workforce development through dedicated training pathways, incentives to retain staff, and integration of YOD-specific expertise into existing professional educations and training programmes in dementia care, including those in nursing, social work, and geriatric medicine.

Furthermore, the expansion of residential care services, including part-time and accomodational arrangements, will allow people to live at home longer. Addressing service gaps, particularly in early-stage support, meaningful daily activities, and services for children of individuals with YOD can help to improve the quality of life and reduce caregiver burden. Finally, systematic dissemination of best practices at the national and international levels can strengthen knowledge exchange, reduce stigma, and inform service development across healthcare systems. To reduce stigma, it is important to raise awareness through concrete strategies such as public awareness campaigns, workplace training for employers, and educational programmes for healthcare professionals. These initiatives can foster earlier recognition of YOD, support social inclusion, and reduce barriers in the access of appropriate services. Collectively, these measures can contribute to more inclusive, personalised care and help reduce regional disparities in access.

### International Implications

By examining how the Netherlands has developed specialised YOD care, international stakeholders, including policymakers, healthcare providers, and researchers, can draw lessons to inform strategies for service development and integration. Countries with decentralised healthcare systems may particularly benefit from adopting elements of the Dutch model for YOD-specialised care. National collaboration combined with regional implementation has facilitated coordination, knowledge sharing, and greater consistency in service delivery. The Dutch hub-and-spoke model, in which regional organisations are supported by the Young-Onset Dementia Knowledge Centre at the national level, may offer a useful template for strengthening equity and innovation in other contexts.^22^

### Future Research

To build on these findings, future studies should include mainstream healthcare organisations that also provide care and support for individuals with YOD. This would provide a more comprehensive understanding of how YOD care is integrated into mainstream dementia care services and where gaps may still exist. Additionally, studies should examine factors associated with optimal service delivery and organisation of such services across regions to identify effective models of care.

Conducting international comparative studies, particularly in countries with decentralised or integrated care systems would help to compare effective service models. Also, it would be interesting to explore how resources can be allocated between mainstream dementia services and YOD-specialised services, particularly in light of the apparent insufficient availability of YOD-specialised services. A better understanding of this distribution could help inform more equitable and needs-based strategies in service delivery. In this context, it is also important to consider how collaboration between mainstream and YOD-specialised healthcare organisations might enhance overall capacity and enable the delivery of tailored care, even in the absence of a fully developed YOD-specific infrastructure.

Finally, including the perspectives of persons with YOD and their carers in these studies is important to gain a more comprehensive understanding of unmet needs within the current delivery and management of care. This can contribute to the alignment of future service development and delivery systems with their lived experiences and preferences.

## Conclusion

While the Netherlands has made notable progress in developing YOD-specialised services, many individuals with YOD remain underserved. Addressing service gaps, improving accessibility, and fostering international knowledge exchange are essential steps towards more equitable and effective care. Moving forward, targeted policy actions and further research are needed to ensure that YOD-specialised services continue to adapt and evolve in response to growing and changing needs, both nationally and internationally.

## Supplemental Material

sj-docx-1-his-10.1177_11786329251388775 – Supplemental material for Exploring the Delivery and Management of Specialised Post-Diagnostic Care and Support in Young-Onset Dementia: A Cross-Sectional Study Supplemental material, sj-docx-1-his-10.1177_11786329251388775 for Exploring the Delivery and Management of Specialised Post-Diagnostic Care and Support in Young-Onset Dementia: A Cross-Sectional Study by Sophie van Westendorp, Cynthia Hofman, Merwin Mortier, Britt Appelhof, Paula Gerring, Raymond Koopmans and Christian Bakker in Health Services Insights
