# Supplementary material for: Exploring the Delivery and Management of Specialised Post-Diagnostic Care and Support in Young-Onset Dementia: A Cross-Sectional Study
Source: Health Serv Insights. 2025 Nov 4;18:11786329251388775. doi: 10.1177/11786329251388775 (PMC12586858; doi:10.1177/11786329251388775)
Supplement: sj-docx-1-his-10.1177_11786329251388775 – Supplemental material for Exploring the Delivery and Management of Specialised Post-Diagnostic Care and Support in Young-Onset Dementia: A Cross-Sectional Study [file sj-docx-1-his-10.1177_11786329251388775.docx]

# YOD Self-scan

Domain 1 The delivery of specialised post-diagnostic services
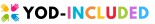


(1/3)

## This domain includes questions on services delivered by the healthcare organisation.

Number

Question

Response

1. **How many individuals with young-onset dementia, on average, use case management**

**from the organisation?** *If this care is not provided, please enter 0. If you and your colleagues do not know or cannot estimate this number, please enter 99999.*

individuals

1. **How many individuals with young-onset dementia, on average, use day care treatment**

Supplementary Material S1

**from the organisation?** *If this care is not provided, please enter 0. If you and your colleagues do not know or cannot estimate this number, please enter 99999.*

individuals

1. **How many individuals with young-onset dementia, on average, use day care from the organisation?** *If this care is not provided, please enter 0. If*

*you and your colleagues do not know or cannot estimate this number, please enter 99999.*

individuals

How many individuals with young-onset

1. **dementia, on average, use permanent residential**

**care from the organisation?** *If this care is not provided, please enter 0. If you and your colleagues do not know or cannot estimate this number, please enter 99999.*

1. **How many individuals with young-onset dementia, on average, use other types of care**

**and support from the organisation?** *If this care is not provided, please enter 0. If you and your colleagues do not know or cannot estimate this number, please enter 99999.*

individuals

individuals

- 1. **If so, what type of care or support does this concern?**

1. **How many YOD-specialised case managers are employed by the organisation?** *If this care is not provided, please enter 0. If you and your colleagues do not know or cannot estimate this number, please enter 99999.*

case managers

6.1

6.2

If so, how many hours per week, on average, offer these case managers to one individual with young-onset dementia?

**If so, what is the average waiting time within the organisation in weeks?** By this, we mean the time between the moment of referral and the first contact with a case manager.

Yes


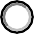

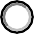


hours per week

weeks

6.3

If so, are case manageres involved when an individual moves to a nursing home?

No, but in development


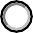
 No


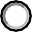
Unknown / prefer not to say

1. **How many places, on average, offers the**

organisation for YOD-specialised day care

**treatment?** *If this care is not provided, please enter 0. If you and your colleagues do not know or cannot estimate this number, please enter 99999.*

places per week

7.1

7.2

**If so, what is the average waiting time for YOD-specialised day care treatment within the organisation in weeks?** By this, we mean the time between the moment of referral and the first contact with day care treatment.

If so, what is the estimated distance from the day care treatment to the visiting individual with young-onset dementia who lives the furthest away?

Supplementary Material S1

If so, are the activities within the

Yes

weeks

kilometers

7.3

organisation's day care treatment groups tailored for individuals with young-onset dementia?


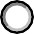
 No, for individuals with dementia in general


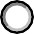

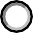
 No, but in development


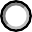

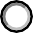


No

Unknown / prefer not to say

7.4

7.5

7.6

If so, what is the composition of the day care treatment groups within the organisation that are focused on individuals with young-onset dementia?

If so, on average, how many individuals with young-onset dementia in a day care treatment group live at home?

If so, on average, how many individuals with young-onset dementia in a day care treatment group live in a nursing home?


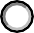
 The group consists solely of individuals with young-onset dementia


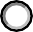
The group consists of all individuals with dementia


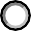
 Other, namely....................

........................................

individuals

individuals

Please proceed to the next page.

# YOD Self-scan

Domain 1 The delivery of specialised post-diagnostic services
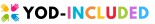


(2/3)

## This domain includes questions on services delivered by the healthcare organisation.

Number

Question

Response

1

**How many places, on average, offers the**

**organisation for YOD-specialised day care?**

places per week

*If this care is not provided, please enter 0. If you and your colleagues do not know or cannot estimate this number, please enter 99999.*

1.1 **If so, what is the average waiting time for YOD- specialised day care in weeks?** *By this, we mean the time*

*between the moment of referral and the first contact with day care.*

Supplementary Material S1

weeks

1.2

1.3

1.4

If so, what is the estimated distance from the day care to the visiting individual with young- onset dementia who lives the furthest away?


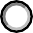

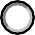
If so, are the activities within the organisation's day care groups tailored for individuals with young-onset dementia?


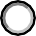

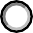

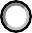


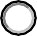
If so, what is the composition of the YOD- specialised day care groups?


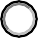

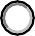


kilometers

Yes

No, for individuals with dementia in general

No, but in development

No

Unknown / prefer not to say

The group consists solely of individuals with young-onset dementia

The group consists of individuals with dementia

Other, namely....................

........................................

1.5

If so, on average, how many individuals with young-onset dementia in a day care group live at home?

individuals

1.6

2

2.1

If so, on average, how many individuals with young-onset dementia in a day care group live in a nursing home?

**How many rooms, on average, offers the organisation for YOD-specialised permanent residential services?** *If this care is not provided, please enter 0. If you and your colleagues do not know or cannot estimate this number, please enter 99999.*

If so, how many rooms, on average, are available for YOD-specialised part-time residential care?

individuals

rooms

rooms

2.2

2.3

2.4

2.5

If so, how many rooms, on average, are available for YOD-specialised accomodational residential care?

**If so, what is the average waiting time for YOD-specialised permanent residential services?** By this, we mean the time between the moment of referral and the moment of admission.

If so, how many residents with young-onset dementia in the organisation have acccess to YOD-specialised day care?

Supplementary Material S1

If so, how many residents with young-onset dementia in the organisation have acccess to YOD-specialised day care?

rooms

weeks

Yes


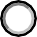

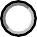

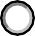

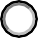


No, but in development

No

Unknown / prefer not to say

Yes


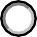

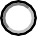

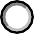

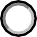


No, but in development

No

Unknown / prefer not to say

1. **Does the organisaton offer any other YOD-specialised services?**
   1. **If so, how many places of this services are available?**


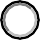
 Yes, namely.........................

.......................................


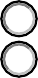
No

Unknown / prefer not to say

Please proceed to the next page.

#
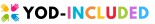
YOD Self-scan

Domain 1 The delivery of specialised post-diagnostic services

(3/3)

## This domain includes questions on services delivered by the healthcare organisation.

Number Question

1. **Which type(s) of services for carers of individuals with young-onset dementia offers the organisation?** *Multiple answers are possible.*

Response


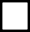
 Support by a case manager


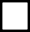
 Individual guidance at home


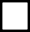
 E-health, for example ''Partner in Balance''
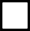
 Courses or trainings


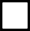
 Peer support or meeting areas


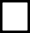
 Coach


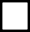
 Care point for carers


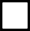
 (behavioural) interventions at home


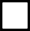
 Other, namely .................................

......................................................

None

1. **Are agreements or work protocols within the organisation on how the service provision for individuals with young-onset dementia and**

Supplementary Material S1

**their carers should be structured?** *For example, this could include agreements or work protocols on the minimum level of care your organisation must provide.*

1. **Does the local government organise YOD- specific activities?**

Yes, completely on paper Yes, not formalised

Yes, partly on paper


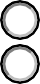

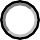

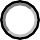


Yes, partly on paper and in the process of full formalisation


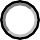
 No, and not in the near future


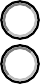


Not yet, but planned for the near future

Unknown / prefer not to say

Yes

No, but in development

No

Unknown / prefer not to say

1. **Are there gaps in the current service provision of young-onset dementia in the organisation?**
2. **Please explain if there is any additional information regarding the organisation's service provision of young-onset dementia.**


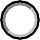
 Yes, namely.....................................


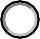
...................................................

No

This is the end of domain 1. Please proceed to domain 2 The organisation of specialised post-diagnostic services

# YOD Self-scan

Domain 2 The management of specialised post-diagnostic services
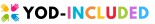


## (1/2) This domain includes questions regarding how the healthcare organisation manages YOD care.

Number Question Response

1. **Describe briefly how the governance structure for YOD-specialised services is structured within the organisation?** *For example, whether it consists of one or multiple business units, the management approach, how exchanges between different units take place, and how aspects such as innovation and development are ensured.*
2. **How many municipalities are, on average, served by the organisation for YOD-specialised services?**

Supplementary Material S1

1. **Which employees with one of the following YOD-specific trainings are employed within the organisation?**

municipalities

3.1

YOD-specific training for caregivers and nurses level 3-4 (*OppStap)*

How many?

YOD-specific case management *(InHolland en Windesheim)*

employees

3.2

3.3

How many?

Interprofessional YOD-specific traning *(Radboudumc Health Academy)*


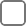


How many?

employees

employees

Training Focussing - for the behavioural variant of frontotemporal dementia *(Waalboog / UKON)*

3.4

3.5

How many?

Intern YOD-specific training

How many?

employees

employees

Other, namely .................................................................................................................

3.6

How many?

None

employees

1. **Are the number of employers with a YOD-specific training employed by the organisation sufficient?**


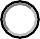
 Yes


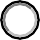
 No, because.............................

............................................

## Please proceed to the next page.

1. **Are there sufficient YOD-specific training opportunities within the organisation?**

Yes

No*,* namely*..............................*


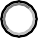

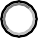


*.............................................*

1. **Is care and support for individuals with young- onset dementia provided by multidisciplinary teams within the organisation?**
   1. **If so, which disciplines are represented in this multidisciplinary team?**

SSuupppplleemmeennttaarryy MMaatteerriiaall SS31

Yes

No, but in development


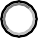

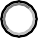

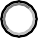

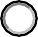


No

Unknown / prefer not to say

- 1. **If so*,* how often, on average, does this multidisciplinary team convey per month?**

times per month

Please proceed to the next page.

#
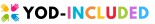
YOD Self-scan

Domain 2 The management of specialised post-diagnostic services

(2/2)

## This domain includes questions regarding how the healthcare organisation manages YOD care.

Number Question Response

*Choose one of the following:*

1 **Are there agreements or work protocols on how the organisation of services for individuals with young-onset dementia and their carers should be structured?** *For example, this could include agreements or work protocols on which disciplines should be involved in the service provision for young-onset dementia.*

- 1. **If so*,* are there agreements or work protocols on how to proceed when YOD- specialised services are unavailable or when service capacity is insufficient?**

Supplementary Material S1

*For example, this could include agreements or work protocols on how to proceed when a YOD-specialised case manager is unavailable.*

How will the organisation proceed

- - - Yes, completely on paper
    - Yes, not formalised
    - Yes, partly on paper
    - Yes, partly on paper and in the process of full formalisation
    - No, and not in the near future
    - Not yet, but planned for the near future
    - Unknown / prefer not to say

*Choose one of the following:*

- - - Yes, completely on paper
    - Yes, not formalised
    - Yes, partly on paper
    - Yes, partly on paper and in the process of full formalisation
    - No, and not in the near future
    - Not yet, but planned for the near future
    - Unknown / prefer not to say

Referral to an organisation without YOD-

**when YOD-specialised services are unavailable or when service capacity is insufficient?** *Multiple answers are possible.*

specialised services

Referral to an organisation with YOD- specialised services

Maintain a waiting list

Other, namely....................................

- 1. **If so, are there agreements or work protocols on involving case managers during the transition to residential care?**
  2. **If so, are there agreements or work protocols on how individuals with young- onset dementia access day care treatment?**

*For example, this includes agreements or work protocols on transportation arrangements such as pick-up or drop-off, or a maximum travel distance*

...........................................................

*Choose one of the following:*

- Yes, completely on paper
- Yes, not formalised
- Yes, partly on paper
- Yes, partly on paper and in the process of full formalisation
- No, and not in the near future
- Not yet, but planned for the near future
- Unknown / prefer not to say

*Choose one of the following:*

- Yes, completely on paper
- Yes, not formalised
- Yes, partly on paper
- Yes, partly on paper and in the process of full formalisation
- No, and not in the near future
- Not yet, but planned for the near future
- Unknown / prefer not to say
  1. ​
  2. ​

**If so, are there agreements or work protocols on how individuals with young- onset dementia access day care?** *For example, this includes agreements or work protocols on transportation arrangements such as pick-up or drop-off, or a maximum travel distance.*

**If so, are there agreements or work protocols on the transfer process when individuals with young-onset dementia transition to resdiential care?** *For example, this includes information that must be shared before or during the transition.*

*Choose one of the following:*

- Yes, completely on paper
- Yes, not formalised
- Yes, partly on paper
- Yes, partly on paper and in the process of full formalisation
- No, and not in the near future
- Not yet, but planned for the near future
- Unknown / prefer not to say

*Choose one of the following:*

- Yes, completely on paper
- Yes, not formalised
- Yes, partly on paper
- Yes, partly on paper and in the process of full formalisation
- No, and not in the near future
- Not yet, but planned for the near future
- Unknown / prefer not to say

1. **Are individuals with young-onset dementia and/or their carers involved in service design and provision?** *For example, through a client council.*
   1. **If so, when are individuals with young-onset dementia and/or their carers invited to provide their input?**


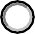
 Yes


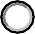
 No, but in development


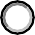
 No


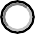
Unknown / prefer not to say

Supplementary Material S1

If so, how are individuals with young-onset dementia and/or their carers invited to provide their input?

1. **What achievements has the organisation made by providing YOD-specialised services?**
2. **Are the components within the care and support for young-onset dementia that the organisation would like to add to its current structure?**
3. **Please explain if there is any additional information regarding the organisation's governance structure for young-onset dementia.**


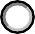
 Yes, namely...............................

..............................................


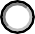
No

This is the end of the YOD self-scan
